# Genistein Inhibition of Topoisomerase IIα Expression Participated by Sp1 and Sp3 in HeLa Cell

**DOI:** 10.3390/ijms10073255

**Published:** 2009-07-22

**Authors:** Najing Zhou, Yunli Yan, Wenling Li, Yanling Wang, Lifen Zheng, Shuo Han, Yongxin Yan, Yunzhi Li

**Affiliations:** 1 Cell Biology Division, Institute of Basic Medicine, Hebei Medical University, Shijiazhuang 050017, Hebei, China; E-Mails: xiaoban8470@126.com (N.J.Z.); liwelling@163.com (W.L.L.); wangyanling200309@163.com (Y.L.W.); lituoxin@sohu.com (L.F.Z.); yongxin604@163.com (Y.X.Y.); 2 Chang’an District Hospital, Shijiazhuang 050017, Hebei, China; E-Mail: liva@tom.com (Y.Z.L.)

**Keywords:** genistein, topoisomerase II-α, specificity protein 1 (Sp1), specificity protein 3 (Sp3)

## Abstract

Genistein (4′, 5, 7-trihydroxyisoflavone) is an isoflavone compound obtained from plants that has potential applications in cancer therapy. However, the molecular mechanism of the action of genistein on cancer cell apoptosis is not well known. In this study, we investigated the effect of genistein on topoisomerase II-α (Topo IIα), an important protein involved in the processes of DNA replication and cell proliferation. The results revealed that inhibition of Topo IIα expression through the regulation of Specificity protein 1 and Specificity protein 3 may be one of the reasons for genistein’s induction of HeLa cell apoptosis.

## 1. Introduction

Genistein is an isoflavone compound obtained from plants which has many physiological functions such as estrogen action, anti-oxidation, mutation prevention, anti-infection, prevention and therapy of heart-cerebrovascular disorders, etc. [1–3], which suggest its usage in health care.

Topoisomerase II (Topo II) is a ubiquitous nuclear protein catalyzing the reaction of breakage and relinking of DNA, which plays an important role in maintenance of DNA topology required for DNA replication, transcription, and recombination of cellular genes. The two Topo II isoforms, Topo IIα and Topo IIβ, are 170 kDa and 180 kDa proteins, respectively, and have different roles in regulating cellular function. Topo IIα shows cell-cycle specific expression during the S and G_2_/M phases and is essential for chromosome condensation. Topo IIα is closely related to the occurrence, development, diagnosis, prognosis and treatment of cancer. Otherwise, Topo IIβ is associated with non-proliferating function and believed to maintain the integrity of nuclear chromatin [4,5].

In recent years, Topo II has become a popular target for cancer chemotherapy treatments. Genistein can inhibit a wide range of cancer cells, and the mechanism of genistein cytotoxicity is considered to involve an inhibitory effect on Topo II [6–9]. It is believed that genistein can bind to and stabilize the topoisomerase-DNA complex, resulting in DNA strand breaks and inducing cell apoptosis [10–13]. However, the molecular events of genistein inhibiting Topo II expression at the gene transcription level have not been clearly understood. The aim of the present study is to investigate the effect of genistein on Topo IIα gene expression and to determine whether the transcription factors Sp1 and Sp3 have a role in the regulation of Topo IIα expression in HeLa cells.

## 2. Results

### 2.1. MTT Assay for Genistein Inhibition of HeLa Cell

**Figure 1.**
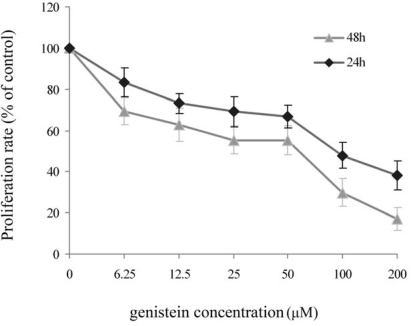
The proliferation rate of genistein on HeLa cells measured by MTT assay. HeLa cells were incubated with different concentrations of genistein for 24 h or 48 h. Cell proliferation rate was determined by comparing genistein treated cells with the control cells. Results showed genistein inhibited HeLa cell proliferation in a dose and time dependent manner. Data were shown as mean ± SD of three representative experiments performed in triplicate.

The effect of genistein on HeLa cell growth was assessed using the MTT assay to measure the cell viability and calculate the half inhibition concentration (IC_50_) of genistein on the cells. Results showed that genistein inhibited HeLa cell growth in a dose and time dependent manner (Figure 1). The IC_50_ value of genistein on HeLa cell was calculated at 126 μM for 24 h and 75 μM for 48 h. In the subsequent experiments, the 48 h IC_50_ value of genistein, 75 μM, was used to treat HeLa cells.

### 2.2. Observation of the Nuclear Morphological Changes

**Figure 2.**
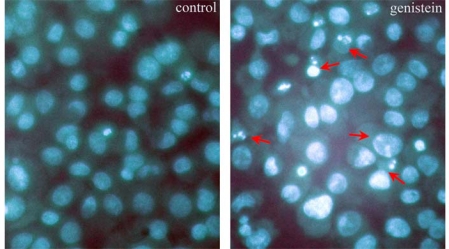
The nuclear morphological changes of the cells were visualized using a fluorescence microscope (original magnification ×400) with Hoechst 33258 staining. The apoptotic cells were characterized by chromatin fragments (arrows).

To verify whether the decreased cell viability of HeLa cells treated with genistein was related to apoptosis, Hoechst 33258 staining was used to detect the nuclear morphological changes. Results showed that HeLa cells treated with genistein for 48 h had undergone remarkable morphological changes. The control cells were well spread flatly and appeared uniform in chromatin density, while genistein treated cells displayed many examples of chromatin fragments or nuclear debris, which could be identified as apoptotic cells (Figure 2), and the ratio of apoptotic cells obviously increased in genistein treated cells (18.34 ± 2.35%) compared to that of control groups (1.52 ± 0.56%). The data shown are means ± SD from three independent experiments. A significant difference (*P*<0.01) was observed between the groups.

### 2.3. Cell Cycle Distribution Revealed by Flow Cytometry

**Figure 3.**
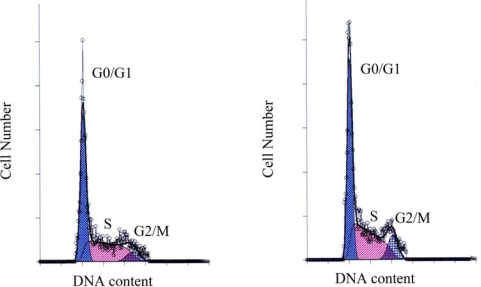
The effect of genistein on HeLa cell cycle distribution revealed with flow cytometry. HeLa cells treated with 75 μM genistein for 48 h were arrested at G_2_/M phase. Data were shown as mean ± SD of three independent experiments performed in triplicate.

After HeLa cells were exposed to 75 μM genistein for 48 h, cell cycle distribution was examined with flow cytometry. Results showed that the percentage of G_2_/M cells increased significantly, while the percentage of G_0_/G_1_ and S cells have no significant difference with that of control group, which suggesting that the cell cycle was arrested at G_2_/M phase (Figure 3, Table 1).

### 2.4. Apoptosis Revealed by Flow Cytometry Using Annexin V/PI Double-staining

**Figure 4.**
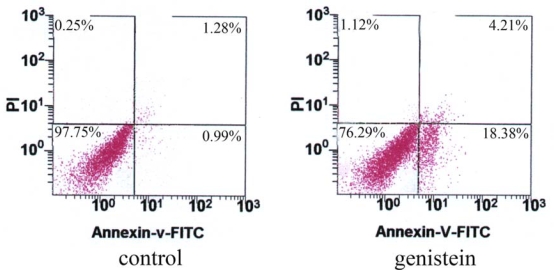
Cell apoptosis measured by annexin V and PI staining with flow cytometry. The intact cells (the lower left quadrant), early apoptotic cells (the lower right quadrant), and late apoptotic cells (the upper right quadrant) were analyzed. The percentage of apoptotic cells significantly increased after genistein treatment. Data were expressed as mean ± SD of three independent experiments (*P*<0.01 vs control).

To assess the capability of genistein inducing HeLa cell apoptosis and distinguish different types of cell death, HeLa cells were double-stained with annexin V and propidium iodide (PI), and analyzed by flow cytometry. This assay was performed to differentiate between intact cells (Annexin-V−/PI−), early apoptotic (annexin V + /PI −) and late apoptotic/necrotic (annexin V + /PI +) cells. The percentage of apoptotic cells was 22.16 ± 2.85% after exposure to genistein for 48 h, while that of control cells was only 2.20 ± 0.62% (Figure 4).

### 2.5. Results of Topo IIα, Sp1 and Sp3 mRNA Expression with Reverse Transcriptase Polymerase Chain Reaction (RT-PCR)

**Figure 5.**
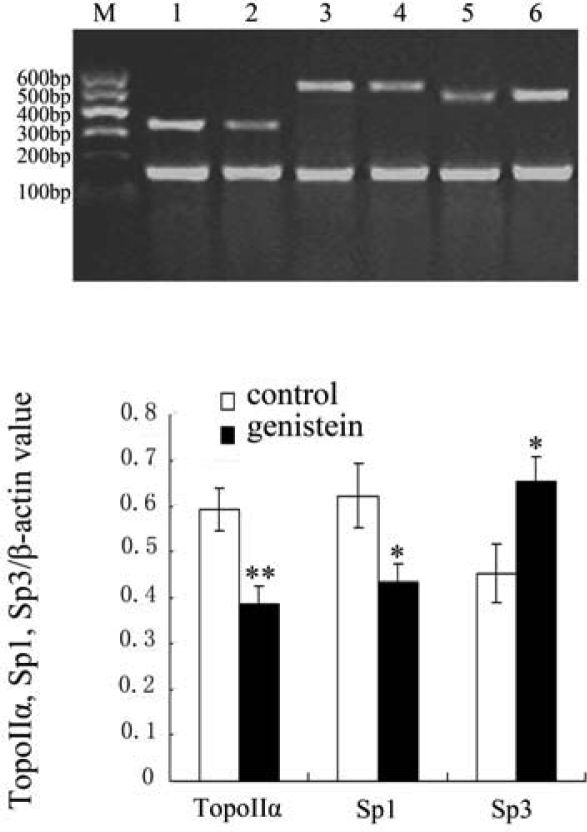
Genistein modulation on the mRNA expression of Topo IIα, Sp1 and Sp3 revealed by RT-PCR. The mRNA expression levels were normalized to that of β-actin. M: marker; Lane 1, 3, 5: control group; Lane 2, 4, 6: genistein treatment group (HeLa cells were treatment with 75 μM genistein for 48 h). Lane 1, 2: Topo IIα; Lane 3, 4: Sp1; Lane 5, 6: Sp3. Data were presented as mean ± SD for three independent experiments (*P<0.05, **P<0.01 vs control).

To investigate the effect of genistein on Topo IIα expression at gene transcription level, HeLa cells were treated with genistein for 48 h and the expression of Topo IIα, Sp1 and Sp3 mRNA in HeLa cells were examined with RT-PCR. Results showed that genistein down-regulated Topo IIα and Sp1 mRNA expression, while Sp3 mRNA expression was up-regulated in HeLa cells (Figure 5).

### 2.6. Western Blot Analysis of Topo II α, Sp1 and Sp3 Protein Expression

**Figure 6.**
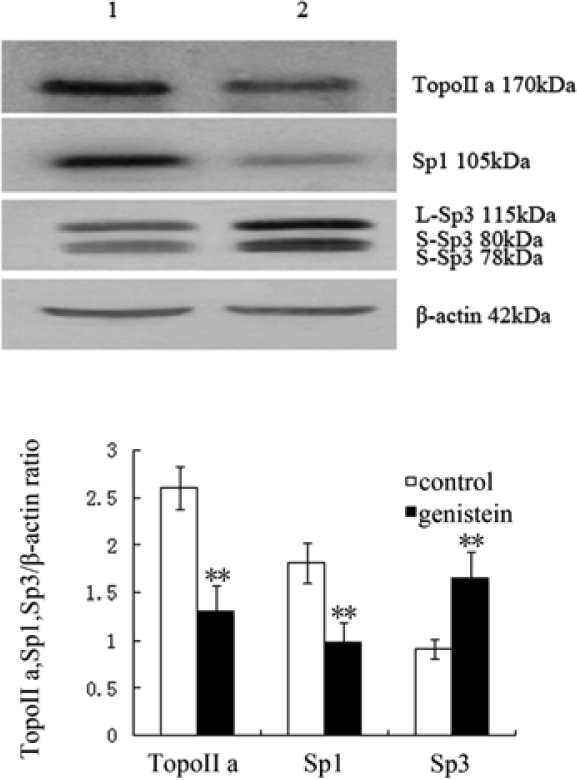
Genistein effects on Topo IIα, Sp1 and Sp3 protein expression revealed by western blot. The protein expression levels were normalized to β-actin. Lane 1: control group, Lane 2: genistein group (HeLa cells were treated with 75 μM genistein for 48 h). Data were presented as mean ± SD for three independent experiments (**P<0.01 vs control).

We examined Topo IIα, Sp1 and Sp3 protein expression in HeLa cells after treatment with genistein by Western blot assay. Results showed that genistein down-regulated Topo IIα and Sp1 protein expression, while Sp3 protein expression was up-regulated in HeLa cells (Figure 6).

### 2.7. The Occupancy of Sp1 and Sp3 Proteins at GC1 and GC2 of Topo II α Promoter Regions by ChIP

**Figure 7.**
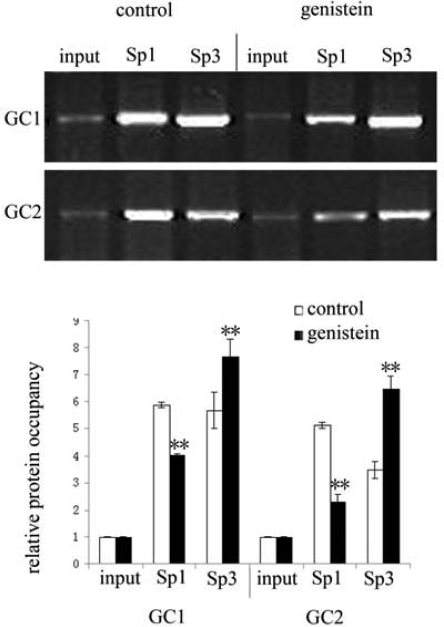
The effect of genistein on the relative occupancy of Sp1 and Sp3 at GC1 and GC2 boxes of Topo IIα promoter revealed by ChIP assay. HeLa cells were treated with 75 μM genistein for 48 h. The corresponding gel photograph showed the PCR products of Sp1 or Sp3 binding to GC1 and GC2 boxes. The relative occupancy was quantified densitometrically in comparision to input (**P<0.01 vs control).

There are two GC boxes, GC1 and GC2 in the Topo IIα promoter, which the transcription factors Sp1 and Sp3 can bind to. To assess the role of Sp1 and Sp3 in the inhibitory effect of genistein on Topo IIα expression, we analyzed their binding ability to the two GC boxes by ChIP experiment. Results showed that Sp1 occupancy was reduced and Sp3 occupancy was elevated in cells treated with genistein compared to controls for both GC1 and GC2 (Figure 7).

## 3. Discussion

To date, growing evidence suggests that genistein has the ability to inhibit cancer cell proliferation and induce cell apoptosis through regulation of biological molecules, such as the estrogen or androgen receptor, protein-tyrosine kinase, NF-kappa B, Akt and MAPK pathways [14–17]. It has been demonstrated that genistein can induce cell cycle G_2_/M arrest and cell apoptosis in many kinds of human cancer cells including neuroblastoma, breast, colon, and lung cancer cells. Recent progress in genistein anti-cancer studies has proven that cell apoptosis induced by genistein is correlated with the expression of a number of genes including those encoding p53, p21WAF1, Caspases, Bax, Cdc25C, Cyclin B1, Bcl-2 [16,18,19]. Furthermore, it was found that genistein inhibits cancer cells through modulating the expression of certain transcription factors, such as STAT-3, Nrf1, Nrf2, AP-1 and CREB [20].

Beyond these properties, genistein has been shown to be potent Topo II poison stabilizing the Topo II protein-DNA cleavable complex, which is similar to the action of many anticancer agents commonly used in chemotherapy, such as etoposide, mitoxantone and amsacrine. It is thought that these drugs, the so-called topoisomerase poisons, can block the ligation step of enzymatic reaction, generating single and double stranded breaks that harm the integrity of the genome. Introduction of these breaks subsequently leads to apoptosis [11–13]. Till now, the mechanism of genistein reaction with the Topo II protein was clear, but whether the drug could directly affect Topo IIα gene expression was unknown.

In the present study, we found that genistein inhibited HeLa cell growth in a dose and time dependent manner. Treatment of HeLa cells with 75 μM genistein (IC_50_) for 48 h caused G_2_/M arrest and apoptosis, which were consistent with the previous findings of genistein on other cancer cells [7,16,19,21]. Additionally, the present study also showed that genistein reduced Topo IIα expression at both mRNA and protein levels in HeLa cells. These results suggest that the drug could inhibit Topo IIα expression at the transcriptional level. Thus, variation of Topo IIα expression may play an important role in genistein- induced HeLa cell apoptosis.

It is known that the human Topo IIα gene promoter has five functional CCAAT boxes and two GC boxes. The two GC boxes, GC1 and GC2 are located at the proximal and distal region of the Topo IIα gene promoter, respectively. Sp1 and Sp3 can bind with similar affinities to both GC boxes by three zinc-fingers [22–25]. It is believed that Sp1 and Sp3 have different functions in regulating Topo IIα gene expression. Sp1 acts mainly as a transcriptional activator, while Sp3, consisting of three subtype proteins of 115 kDa, 80 kDa, 78 kDa, respectively, can act as a transcriptional repressor binding the GC boxes competitively with Sp1 [26,27]. Recently, it has been shown that the GC-rich sequence is necessary for the effect of genistein on metal-binding protein, metallothionein IIA expression in human intestinal Caco-2 cells [28]. Moreover, genistein can block the stimulation of VEGF gene transcription by all trans-retinoic acid through Sp1 and Sp3 sites in a human bronchioloalveolar carcinoma cell [29]. Thus, it is likely that genistein may be able to regulate Topo IIα gene expression through Sp1 and Sp3.

To determine if Sp1 and Sp3 were involved in genistein inhibition of Topo IIα, the present study examined their expression in HeLa cells. Results revealed that genistein could down-regulate Sp1 expression, and up-regulate Sp3 expression simultaneously. The ChIP experiment in this study confirmed that genistein had an influence on Topo IIα expression through alterations in Sp1 and Sp3 binding with Topo IIα promoter. Taken together, the present study suggests that genistein inhibition of Topo IIα expression leading to HeLa cell apoptosis may be mediated by the transcription factors Sp1 and Sp3 although the involvement of other factors cannot be ruled out.

## 4. Experimental Section

### 4.1. Drugs and Reagents

Genistein and Hoechst 33258 were purchased from Sigma (USA). RPMI 1640 medium, newborn calf serum and methylthiazolyl tetrazolium (MTT) were bought from Gibco (USA). RNase, Trizol and avian myeloblastosis virus (AMV) reverse transcriptase were purchased from Promega (USA). All PCR primers were synthesized by Sbsbio (Beijing, China). The primary polyclonal mouse anti-Topo II α, Sp1, Sp3, β-actin antibodies, Annexin V-FITC and the protein A/G plus-agarose beads were from Santa Cruz Biotechnology (USA).

### 4.2. Cell Culture

HeLa cells were cultured in RPMI 1640 medium supplemented with 10% newborn calf serum, 10,000 IU/L penicillin and 10 mg/L streptomycin in the environment of 37 °C, 5% CO_2_.

### 4.3. MTT Assay for Genistein Inhibition of HeLa Cell

Logarithmically growing HeLa cells were seeded at a density of 10^5^ cells per well in 96-well plates, and allowed to adhere for 4 h at 37 °C. Equal volumes of different concentrations of genistein (dissolved in dimethyl sulfoxide, DMSO) were added and cultured for 24 h or 48 h. The control group was given DMSO only. MTT (5 g/L) was added into each well for an additional 4 h incubation at 37 °C. Untransformed MTT was removed by aspiration and formazan crystals were dissolved in DMSO (200 μL per well). The absorbance was read with a microplate reader (Elx800, Bio-Tek, USA) at 490 nm. The cell proliferation rates were calculated according to the following formula: cell proliferation rate (%) = (absorbance of the treated wells/absorbance of the control wells) x 100%. The IC_50_ (50% inhibition concentration) values were calculated by dose–response curves using the Graph Pad Prism soft ware (GraphPad Software Inc, San Diego, CA, USA).

### 4.4. Observation of Nuclear Morphological Changes by Hoechst 33258 Staining

The nuclear morphological changes of the cells were observed and apoptotic cells were identified by Hoechst 33258 staining. Briefly, the cells were seeded on coverslips and incubated for 48 hours with or without genistein. Cells were fixed with 4% paraformaldehyde for 10 min and washed in 0.01 M PBS (pH 7.4). After the nuclear DNA was stained with 10 μg/mL of Hoechst 33258 for another 5 min, cells were observed using a fluorescence microscope (Olympus, Japan). Apoptotic cells characterized by chromatin fragments were counted in five random fields at a magnification of 200 x for each group of cells.

### 4.5. Cell Cycle and Apoptosis Analysis with Flow Cytometry

After treatment with 75 μM genistein for 48 h, 1×10^5^ cells were harvested for each group and washed in PBS. After fixing in cold alcohol, the cells were treated with 100 mg/L RNase in PBS for 1 h, followed by staining with 50 mg/L propidium iodide in PBS. Cell cycle distribution was detected using an ETICS-XL™-flow cytometer (Beckman Coulter, USA). Data were analyzed with MultiCycle AV software (Phoenix Flow Systems, San Diego, CA, USA).

### 4.6. Apoptosis Analysis by Annexin V-FITC/PI with Flow Cytometry

To determine the apoptotic effect of genistein, we incubated HeLa cells with a solution of fluorescein isothiocyanate labelled with Annexin V (AnnexinV-FITC) and PI. Cells were treated with genistein for 48 h. The sample cells were harvested, centrifuged, washed twice with PBS and resuspended in binding buffer diluted 1:10 to 10^6^ cells per mL. The cells were then stained for 10 min with FITC-AnnexinV and PI and recorded using a FACSAria cell sorter. The percentage of intact and apoptotic cells were calculated using Flowjo software (Flowjo Tree Star Inc., Ashland, OR, USA).

### 4.7. RT-PCR for Topo IIα, Sp1 and Sp3 mRNA Expression

For RT-PCR, 5×10^6^ cells treated by 75 μM genistein for 48 h were harvested and washed in PBS. Total RNA was extracted with TRIZOL reagent, the cDNA was synthesized at 37°C for 1h in a 15 μL reaction mixture containing total RNA (2 μg/μL), random primer (500 ng/μL 1 μL, RNase (50 U/μl) 0.5 μL, 5×AMV RT buffer 3 μL, 10 mM dNTP 1.5 μL, MgCl_2_ 0.5μL, AMV reverse transcriptase (300 U/μL) 1 μL, 1‰ DEPC 3.5 μL, then AMV reverse transcriptase were destroyed at 95°C for 5 min.

PCR was carried out in a reaction mixture containing sense and antisense primers (10 μM) 1 μL, 0.2 mM dNTPs 2.5 μL, Red Taq DNA polymerase (1U/μL) 1 μL and cDNA 1 μL in a total volume of 20 μL. Fragments were amplified with the following PCR cycles: 94 °C for 5 min, 35 cycles (25 cycles for β-actin) of 94 °C for 40 s, annealed for 35 s and 72 °C for 35 s, and finally 72 °C for 5 min. The primer sequences, product length and the anneal temperature of the target genes are shown in Table 2. The PCR products were separated by electrophoresis on 1.5% agarose gels and visualized by ethidium bromide staining. The relative expression was quantified densitometrically using an image analysis system (UVP-Lab work 4.60, USA), and calculated according to the reference bands of β-actin.

### 4.8. Western Blot Analysis Topo IIα, Sp1 and Sp3 Protein Expression

After treatment with 75 μM genistein for 48 h, the cells were homogenized in buffer containing 50 mM Tris-HCl, pH 7.6, 150 mM NaCl, 1% Nonidet P-40, 0.1% SDS, 0.5% sodium deoxycholate, 1 mM EDTA, 1 mM phenylmethylsulfonyl fluoride, 2 mg/L aprotinin, 2 mg/L leupeptin, and 0.5 mM dithiothreitol, and centrifuged at 1,500 g for 10 min at 4°C. The same amount of proteins (100 μg each lane) were electrophoresed on 10% polyacrylamide gels and transferred to nitrocellulose membrane. The membranes were blocked for 1 h in 5% nonfat milk in Tris-buffered saline (TBS). Mouse anti-Topo IIα, Sp1, Sp3 and β-actin primary antibodies were added in TBS, the blots were incubated overnight at 4 °C. After washing, they were incubated with HRP-conjugated goat anti-mouse IgG in 10 mL TBS for 1 h at 37 °C. Blots were then treated with a chemiluminescent detection system using the SuperSignal West Pico Substrate and exposed to film. Digital images were captured and quantified using an image analysis system (UVP-Lab work 4.60, USA). The expression levels of target proteins were calculated according to the reference bands of β-actin.

### 4.9. Chromatin Immunoprecipitation (ChIP) Assays Analysis Sp1 and Sp3 Protein Binding to Topo IIα Promoter

The ChIP was done after HeLa cells had been treated with 75 μM genistein for 48 h. Briefly, the cells were cross-linked with 1% formaldehyde for 10 min at 37 °C and then quenched by the addition of 0.125 M glycine. The cells were rinsed in cold PBS and collected by centrifugation, resultant pellets were resuspended in 300 μL of lysis buffer [10 mM potassium acetate, 15 mM magnesium acetate, 0.1 M Tris (pH 7.6), 0.5 mM phenylmethylsulfonyl fluoride, and 100 μg of leupeptin and aprotinin/L], incubated on ice for 20 min. The nuclei were incubated in sonication buffer [1% sodium dodecyl sulfate, 10 mM EDTA, 50 mM Tris-HCl (pH 8.1), 0.5 mM phenylmethylsulfonyl fluoride, and 100 ug of leupeptin and aprotinin/L] on ice for 10 min and sheared by sonication to produce approximate 400 bp fragments. After centrifugation, the supernatant was diluted and 1% used for input. The remainder was incubated with sheared herring sperm DNA (2 g/L), 20 μL pre-immune serum and 45 μL of 50% protein A/G plus-agarose beads for 2 h at 4°C. After centrifugation, 1 μL (200 g/L) Sp1 and Sp3 antibodies were added to the supernatant and incubated overnight at 4°C. Then, 45 μL protein A/G plus-agarose beads were added followed by herring sperm DNA and the mixture was incubated for another 1 h at 4 °C. The beads were harvested and washed. The DNA-protein complex was eluted with 100 μl elution buffer (1% SDS, 0.1 M NaHCO_3_) at room temperature for 10 min. The eluate was heated at 65 °C for 4h to reverse formaldehyde cross-links with 0.5 M NaCl and 10 μg of RNase. After that, the DNA was extracted from the eluate by the phenol/chloroform method and then precipitated with ethanol. The purified DNA was subjected to PCR with primers specific for the Topo IIα promoter region. Spanning two putative Sp1 and Sp3 binding sites respectively, the sequences of the PCR primers used are as follows:

GC1 (proximal): Sense: 5′-ACTCAGCCGTTCATAGGT-3′,
Antisense: 5′-AGCCGCTTCTCCACA-3′;
GC2 (distal): Sense: 5′-GGGGTCTCGCTATGTT-3′
Antisense: 5′-CTGGCTGCTTGGTTG-3′

PCR was carried out as follows: 94 °C for 5 min, 35 cycles of 94 °C for 40 s, annealed at 52 °C for 35 s and 72 °C for 35 s, and finally 72 °C for 5 min. The PCR products (161 bp for GC1 and 202 bp for GC2) were separated by electrophoresis on 1.5% agarose gels and visualized by ethidium bromide staning. The relative occupancy was quantified densitometrically using an image analysis system (UVP-Lab work 4.60, USA) and calculated according to the corresponding bands of input.

### 4.10. Statistics

All experiments were done independently at least three times in triplicate. GraphPad Prism 4 software (GraphPad Software Inc, San Diego, CA, USA) was used for statistical analyses. Data were presented as means ± standard deviation (SD) and analyzed by Student’s two-tailed, unpaired *t*-test. *P* < 0.05 was considered significantly different.

## 5. Conclusions

Our data shows that suppressing Topo IIα expression through Sp1 and Sp3 plays important role in genistein induction of HeLa cell G_2_M arrest and apoptosis.

## Figures and Tables

**Table 1.** Cell cycle distribution tested with flow cytometry (*x̄* ± *s*%).

**Group**	**G_0_/G_1_**	**S**	**G_2_/M**
**Control**	50.64 ± 2.35	42.31 ± 2.66	7.06 ± 0.33
**Genistein**	45.77 ± 4.03	42.40 ± 3.60	11.83 ± 0.61*

* *P<*0.01 *vs* control group.

**Table 2.** The primer sequences, amplicon length and annealing temperatures of target genes.

**Name**	**Primer sequence**	**Length**	**Annealing temperature**
**TopoIIα**	Sense: 5′-TGCCTGTTTAGTCGCTTTC-3′	349bp	50.5 °C
	Antisense 5′-TGAGGTGGTCTTAAGAAT-3′		
**Sp1**	Sense: 5′-TGGTGGGCAGTATGTTGT-3′	550bp	55 °C
	Antisense 5′-GCTATTGGCATTGGTGAA-3′		
**Sp3**	Sense: 5′-TAAGGTGTATTGCGTCTT-3′	516bp	50 °C
	Antisense 5′-GCTATTGGCATTGGTGAA-3′		
**β-actin**	Sense: 5′-CCA ACTGGGACGACAT-3′	135bp	54 °C
	Antisense: 5′-TCTGGGTCATCTTCTCG-3′		
